# Systemic administration of the di-apocarotenoid norbixin (BIO201) is neuroprotective, preserves photoreceptor function and inhibits A2E and lipofuscin accumulation in animal models of age-related macular degeneration and Stargardt disease

**DOI:** 10.18632/aging.103014

**Published:** 2020-04-07

**Authors:** Valérie Fontaine, Elodie Monteiro, Mylène Fournié, Elena Brazhnikova, Thinhinane Boumedine, Cécile Vidal, Christine Balducci, Louis Guibout, Mathilde Latil, Pierre J. Dilda, Stanislas Veillet, José-Alain Sahel, René Lafont, Serge Camelo

**Affiliations:** 1 Sorbonne Université, INSERM, CNRS, Institut de la Vision, Paris 75012, France; 2 Biophytis, Sorbonne Université, Campus Pierre and Marie Curie, Paris 75005, France

**Keywords:** norbixin, retinal function, A2E, AMD, Stargardt disease

## Abstract

Atrophic A\age-related macular degeneration (AMD) and Stargardt disease (STGD) are major blinding diseases affecting millions of patients worldwide, but no treatment is available. In dry AMD and STGD oxidative stress and subretinal accumulation of *N*-retinylidene-*N*-retinylethanolamine (A2E), a toxic by-product of the visual cycle, causes retinal pigment epithelium (RPE) and photoreceptor degeneration leading to visual impairment. Acute and chronic retinal degeneration following blue light damage (BLD) in BALB/c mice and aging of *Abca4^-/-^ Rdh8^-/-^* mice, respectively, reproduce features of AMD and STGD. Efficacy of systemic administrations of 9'-*cis*-norbixin (norbixin), a natural di-apocarotenoid, prepared from *Bixa orellana* seeds with anti-oxidative properties, was evaluated during BLD in BALB/c mice, and in *Abca4^-/-^ Rdh8^-/-^* mice of different ages, following three experimental designs: “preventive”, “early curative” and “late curative” supplementations. Norbixin injected intraperitoneally in BALB/c mice, maintained scotopic and photopic electroretinogram amplitude and was neuroprotective. Norbixin chronic oral administration for 6 months in *Abca4^-/-^ Rdh8^-/-^* mice following the “early curative” supplementation showed optimal neuroprotection and maintenance of photoreceptor function and reduced ocular A2E accumulation. Thus, norbixin appears promising as a systemic drug candidate for both AMD and STGD treatment.

## INTRODUCTION

Age-related macular degeneration (AMD) is the commonest cause of severe visual loss and blindness in developed countries among individuals aged 60 and older [1]. AMD is a major unmet medical need as it is estimated that more than 20 million patients will be affected by 2050 in the US alone. STGD is the most common hereditary macular dystrophy, mostly affecting young patients aged between 6 and 15 years old with a prevalence of 1/8,000-1/10,000 [2, 3]. It has an autosomal recessive mode of inheritance and may lead to registered blindness within the second or third decade of life. STGD is caused by mutations in the *ABCR* gene encoding the ATP-binding cassette gene, subfamily A, member 4 (ABCA4) transporter which is expressed by photoreceptors and retinal pigmented epithelium (RPE) cells and plays an important role in the visual cycle [4, 5]. Polymorphisms of the gene coding for ABCA4 have also been associated with increased risk of developing AMD [6]. Other genetic polymorphisms, especially in the *complement factor H* (*CFH*) gene [7] have also been associated with AMD, but risk factors are mostly linked to age [8, 9] and environmental such as smoking [10]. AMD can either evolve towards neovascular AMD, also called wet AMD, characterized by the growth of new choroidal blood vessels in the subretinal space, or towards geographic atrophy also named dry AMD, characterized by RPE and photoreceptor degeneration. In STGD patients, neovascularization is extremely rare and RPE and photoreceptor atrophy occurs in the vast majority of cases [11]. Thus, despite some differences, dry AMD and STGD share similar pathophysiological mechanisms [11]. In both pathologies, early signs of evolution are characterized by subretinal accumulation of lipids and proteins forming drusen in AMD and flecks in STGD [2, 12]. AMD and STGD evolution are associated with Bruch’s membrane thickening, RPE alterations and ultimately to RPE and photoreceptor degeneration. Rod photoreceptors, responsible for scotopic/mesopic vision (i.e. under dim light conditions) are the first visual cells dying in the retina. The cones mediating colored photopic vision under normal light conditions are essentially preserved until late stages of AMD and STGD [13, 14]. Therefore, both AMD and STGD induce the progressive loss of night vision followed by loss of color vision and central vision [3]. To date, no treatment is available for either STGD [11] or dry AMD [15].

Both drusen in AMD and flecks in STGD contain *N*-retinylidene*-N*-retinylethanolamine (A2E), which is a toxic by-product of the visual cycle [16]. It is formed by the reaction of 2 all*-trans* retinal molecules with phosphatidylethanolamine generating *N*-retinylidene-PE (A2E precursor: A2-PE), as a detoxication mechanism of retinal isomers including all-*trans* and 11-*cis*-retinal [17]. Under normal conditions the ABCA4 protein participates in the elimination of A2-PE from the photoreceptors and inhibition of this clearance increases the accumulation of A2E and all-*trans*-retinal dimer in the RPE [18, 19]. Recently, it has been shown that ABCA4 is also expressed in RPE cells where it would participate in the recycling of retinaldehyde released during proteolysis of rhodopsin in endolysosomes following phagocytosis of photoreceptor outer segments [5]. The authors also demonstrated that A2E accumulates at similar rates in RPE from *Abca4*^-/-^ mice reared under cyclic light or total darkness suggesting that *de novo* bis-retinoids formation within RPE endolysosomes contributes more to lipofuscin build-up than do bis-retinoids formed in outer segment discs during light exposure. Therefore, they propose that the clearance of retinaldehydes from RPE phagolysosomes may be more critical for photoreceptors viability than the clearance of retinaldehydes from outer segment discs [5]. *In vitro*, it has been shown that the combination of blue-light illumination and A2E is toxic for RPE cells; either the ARPE19 cell line or primary porcine RPE cells [20–22]. Indeed, in the presence of blue light and oxygen, A2E undergoes photo-oxidation as evidenced by the appearance of toxic oxygen adducts [23]. It generates small amounts of singlet oxygen and is finally cleaved to small reactive aldehydes, which contribute to its deleterious effects on RPE cells [23]. A2E photo-oxidation products also damage DNA [20, 21] and activate the complement system [24]. Moreover, photosensitization of A2E triggers telomere dysfunction and accelerates RPE senescence [25]. In the absence of illumination, A2E alone affects normal RPE functions by inducing membrane permeabilization and thereby impairing lysosomal function [26]. It also impairs mitochondrial homeostasis and function resulting in the reduction of ATP production [27–30]. Furthermore, high A2E concentrations increase oxidative stress [31] and secretion of inflammatory cytokines by RPE cells *in vitro* [25, 32, 33]. In addition, A2E induces the expression of vascular endothelial growth factor *in vitro* [34] and *in vivo* [32, 35].

A rapid accumulation of A2E and of lipofuscin is observed in 3-month-old *Abca4^-/-^ Rdh8^-/-^* mice [36] and increases in 6-month-old mice [36]. In these mice, progressive A2E accumulation is associated with retinal degeneration and loss of both scotopic and photopic full-field electroretinogram (ERG) responses indicate that both rods and cones are dysfunctional (rod-cone dystrophy) [36, 37]. Indeed, it has been shown that as early as in 3-month-old *Abca4^-/-^ Rdh8^-/-^* mice, the amplitudes of scotopic A and B waves and flicker ERG are reduced [36]. *Abca4^-/-^ Rdh8^-/-^* mice recapitulate most phenotypic retinal alterations observed during STGD and AMD [36, 37], and represent a chronic model of these diseases.

Acute white-light exposure is known to induce apoptosis of photoreceptors in albino mice retinas [38]. The model of light-induced retinopathy is used as an amenable model of AMD and has been used to test several neuroprotective molecules [39].

Norbixin (9’-*cis*-norbixin) is a 6,6’-di-*apo*-carotenoid extracted from annatto (*Bixa orellana*) seeds [40]. Tolerability of norbixin is well known, based on animal and human studies, and supports its use as food additive/dye [41]. Norbixin protects primary porcine RPE cells against phototoxicity induced by A2E and blue-light illumination *in vitro* [22]. Norbixin also reduces the accumulation of A2E by primary porcine RPE cells *in vitro* [22]. We have previously reported that a 3-month supplementation with norbixin in water reduced A2E ocular accumulation *in vivo* in *Abca4^-/-^ Rdh8^-/-^* double-knockout mice [22]. In the same mouse model, the local treatment with norbixin via intraocular injections inhibited retinal degeneration and the loss of full-field ERG, induced following blue-light illumination [22]. In addition, norbixin was neuroprotective against blue-light damage (BLD) in rats [22].

Here we used an acute BLD model in BALB/c mice modified from our previous article [22] and that is used to rapidly study some features of AMD and the chronic model of AMD and STGD in aging *Abca4^-/-^ Rdh8^-/-^* mice to evaluate the protective efficacy of norbixin. In the present article we demonstrate that norbixin administered systemically is neuroprotective against blue-light-induced retinal degeneration in BALB/c mice. Moreover, we report for the first time that 6-month oral supplementation of *Abca4^-/-^ Rdh8^-/-^* double-knockout mice with chow containing norbixin is neuroprotective and partially preserves the function of both rods and cones *in vivo*. Oral supplementation with norbixin also reduces A2E and lipofuscin accumulation in RPE cells. We define the therapeutic window during which oral supplementation with norbixin is the most effective in relation to progressive photoreceptor loss of function and A2E accumulation.

## RESULTS

### Norbixin protects the retina of albino BALB/c mice against blue-light-induced photoreceptor degeneration

**Figure 1:**
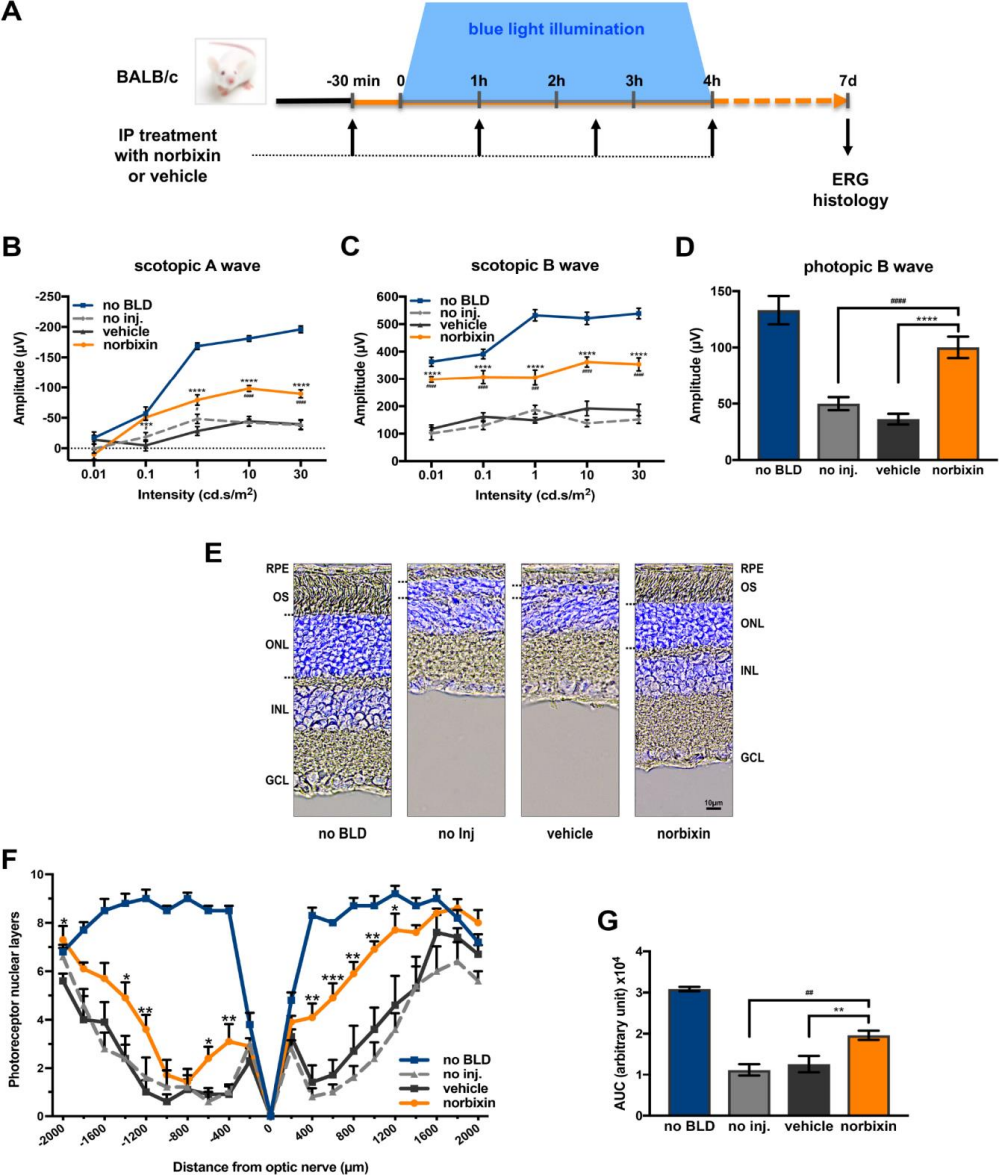
**Effect of norbixin on ERG and retinal phototoxicity after BLD in BALB/c mice.** (**A**) schematic representation of the protocol design. (**B**) Scotopic A wave, (**C**) Scotopic B wave, (**D**) Photopic B wave, ERG recorded 7 days after BLD. (**E**) Representative cryosection pictures showing Hoechst 33342 staining of the retinal cell nuclei one week after BLD. (**F**) Graph showing the number of photoreceptor layers measured along the retina each 200 μm from the optic nerve. (**G**) Histograms showing the area under the curve (AUC) calculated from the photoreceptor layer quantification and used to perform statistical analyses. IP: intra-peritoneal; no BLD: no blue light damage; no inj.: no injection; OS: outer segment; ONL: outer nuclear layer; INL: inner nuclear layer; GCL: ganglion cell layer. Bars represent mean ± s.e.m. with n = 8 per group. ^#^ or *p<0.05, ^##^ or **p<0.01, ^###^ or ***p<0.001, ^####^ or ****p<0.0001 compared to non-injected or to vehicle, respectively (One-way ANOVA, Dunnett's post-test).

To determine whether norbixin could protect the retina via a systemic effect we used a model of BLD in albino BALB/c mice. Norbixin (10 mg/kg of body weight) was injected intraperitoneally 30 minutes prior to BLD and 1, 2.5 and 4 hours after the beginning of exposure to blue light (Figure 1A). Four hours of blue-light exposure induced severe loss of retinal function in vehicle-dosed mice, as measured seven days after exposure by scotopic A wave (Figure 1B), scotopic B wave (Figure 1C) and photopic B wave (Figure 1D) ERGs. In the light-exposed mice, intraperitoneal administration of norbixin maintained 40.4 % of the scotopic A wave and 58.4 % of scotopic B wave amplitudes at a flash intensity of 10 cd.s/m^2^ compared to the non-injected group (*p<*0.0001), whereas there was no effect in the vehicle group. Norbixin treatment also allowed a 60.4 % (*p<*0.001) preservation of the photopic B wave intensity (Figure 1D). The neuroprotective effect of norbixin was confirmed by morphological evaluation of the retina, 7 days following BLD (Figure 1E). Consistently, treatment with norbixin partially protected photoreceptors against degeneration induced by BLD. Indeed, more photoreceptors were preserved in the norbixin group (+43 %; *p*<0.01) than in the non-injected or the vehicle treated groups (Figure 1F and 1G).

### Determination of retinal degeneration, ERG amplitudes and A2E accumulation kinetics in Abca4^-/-^ Rdh8^-/-^ mice of different ages

**Figure 2:**
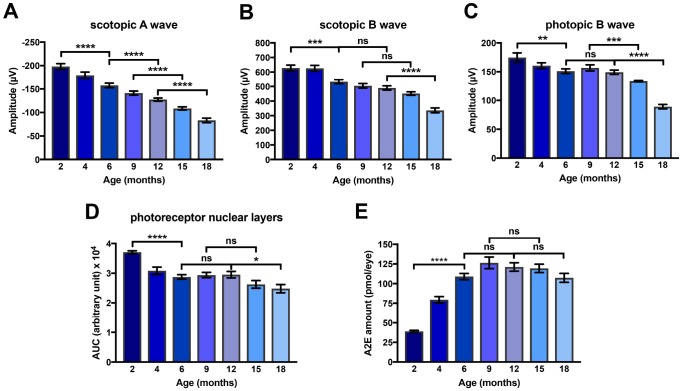
**Kinetics of loss of amplitude of scotopic A and B waves and photopic B wave, of A2E accumulation and of loss of photoreceptor nuclear layers in *Abca4^-/-^ Rdh8^-/-^* mice aged between 2 and 18 months.** (**A**) Scotopic A wave recorded at a flash intensity of 10 cd.s/m^2^ (n = 30-50). (**B**) Scotopic B wave recorded at a flash intensity of 10 cd.s/m^2^ (n = 30-50). (**C**) Photopic B wave recorded at a flash intensity of 30 cd.s/m^2^ (n = 30-50). (**D**) Kinetics of loss of photoreceptor nuclear layers (n = 7-14). (**E**) Kinetics of A2E accumulation (n = 15-40). Bars represent mean ± s.e.m. *p<0.05, **p<0.01, ***p<0.001, ***p<0.0001 (One-way ANOVA, Dunnett's post-test).

We characterized precisely the kinetics of i) photoreceptor degeneration of ii) the progressive loss of visual function and of iii) the accumulation of A2E in eyes of *Abca4^-/-^Rdh8^-/-^* mice in a systematic analysis of this mice model between 2 and 18 months of age. First, in the *Abca4^-/-^Rdh8^-/-^* mouse model we measured scotopic and photopic ERGs. We found that the amplitude of scotopic A and B waves and photopic B wave decreased progressively between 2 and 18 months of age (Figure 2A–2C). The progressive loss of visual function was paralleled by reduction of the number of photoreceptor nuclei (Figure 2D). Interestingly, losses of visual function and of photoreceptor nuclei started early during the course of aging since diminution were significantly different (scotopic A wave *p<*0.0001, scotopic B wave *p<*0.001, photopic A wave *p<*0.01 and photoreceptors loss *p<*0.0001) between 2 and 6 months (Figure 2A–2D). At later time points, a significant alteration of ERG was also observed between 6 and 12 months (scotopic A wave only), 9 and 15 months (scotopic A and photopic B waves), and 12 and 18 months (scotopic and photopic A and B waves). The decrease of photoreceptor layers number occurred in three steps: a strong loss between 2 and 6 months followed by a plateau until 12 months, and a second decrease period between 12 and 18 months (Figure 2D). We then determined ocular A2E accumulation in the eyes of *Abca4^-/-^ Rdh8^-/-^* mice. A2E ocular concentration increased significantly between 2 and 6 months (*p<*0.0001), reached a maximum at 9 months of age followed by a plateau (Figure 2E).

### RPE65, cathepsin D and GFAP immunostaining in Abca4^-/-^ Rdh8^-/-^ mice of different ages

**Figure 3:**
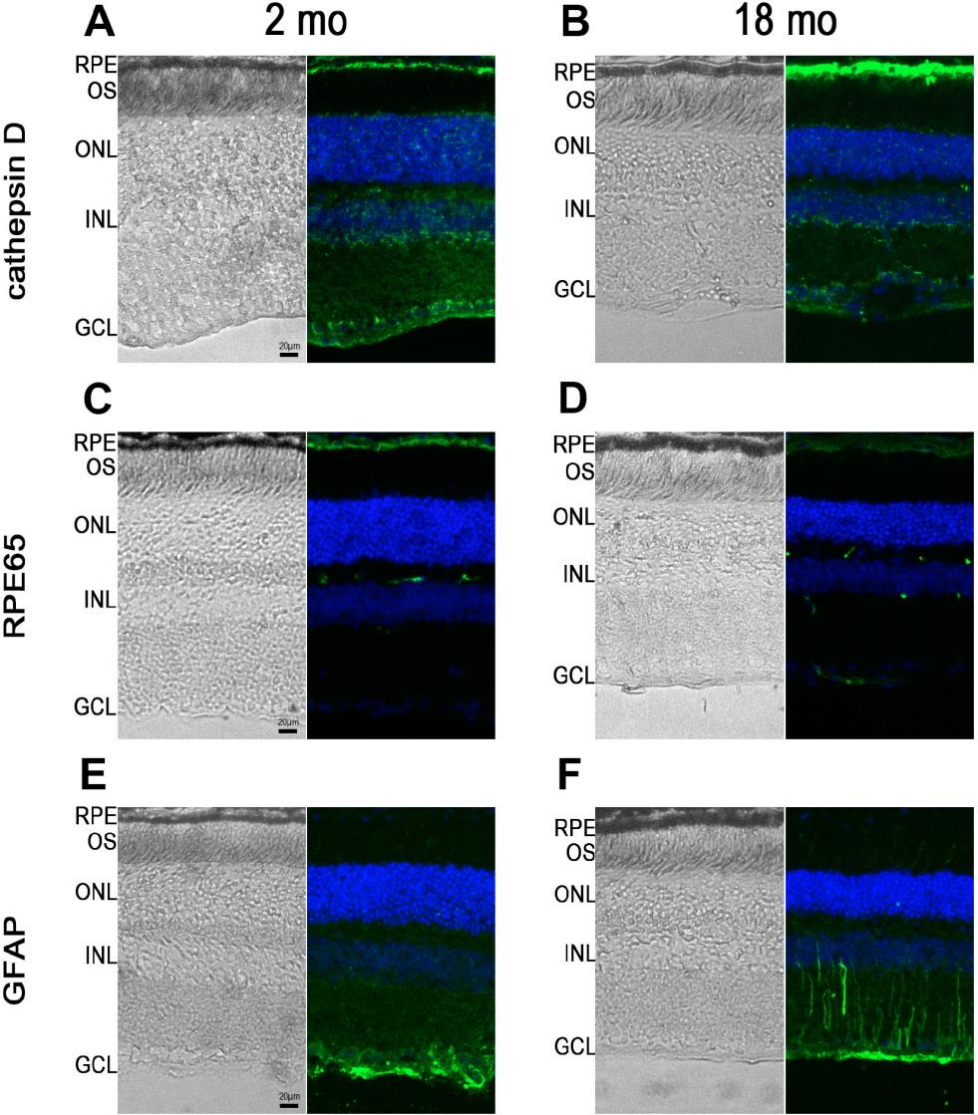
**RPE65, cathepsin D and GFAP immunostaining in *Abca4^-/-^ Rdh8^-/-^* mice of different ages.** Pictures showing retinal cryosections of 2-month-old (**A**, **C**, **E**) and 18-month-old (**B**, **D**, **F**) mice captured in bright field (left pictures) or after immunostaining for cathepsin D (**A**, **B**), RPE65 (**C**, **D**) and GFAP (**E**, **F**). OS: outer segment; ONL: outer nuclear layer; INL: inner nuclear layer; GCL: ganglion cell layer.

Next, we performed immunohistochemistry studies to characterize RPE65 (an enzyme of the visual cycle), cathepsin D, a lysosomal enzyme expressed in RPE, and GFAP, a marker of astrocytes and reactive Müller cells, expression in 2- and 18-month-old *Abca4^-/-^ Rdh8^-/-^ mice*. At 2 months of age, as expected, cathepsin D expression was observed in various retinal cells but was more intense at the level of the RPE (Figure 3A). The same pattern of expression was noted in 18-month-old mice, however we noted that RPE cells appeared thicker both on bright light image and following fluorescent staining (Figure 3B). An Increase in intensity of cathepsin D expression could be detected between young and old mice (Figure 3B). Similarly, in 2-month-old *Abca4^-/-^* *Rdh8^-/-^* mice, RPE layer was thin and RPE cells appeared uniformly immunoreactive for RPE65 (Figure 3C). By contrast, at 18 months, the RPE cells were thicker. In addition, RPE65 expression almost disappeared completely from the apical face. The weak remaining staining appeared mostly basal (Figure 3D). Finally, we performed immunohistochemistry for GFAP that showed that GFAP expression was limited to astrocytes in 2-month-old *Abca4^-/-^ Rdh8^-/-^* mice (Figure 3E), but that in 18-month-old mice, Müller cells were also GFAP positive, which is consistent with retinal stress (Figure 3E). Altogether, we described progressive reduction in ERG amplitudes, increased A2E accumulation, modifications of the RPE layer and apparition of retinal stress which are linked to progressive photoreceptor loss, as the *Abca4^-/-^ Rdh8^-/-^* mice got older. The observations that the kinetics of evolution of these parameters could vary over time, prompted us to test the efficacy of norbixin administered by oral supplementation for 5 to 6 months in mice of various ages. In an attempt to determine the norbixin supplementation therapeutic window, we decided to follow three experimental designs: “preventive”, “early curative” and “late curative” supplementations in which norbixin administration started at 1.5 months, 9 months, and 12 months of age, respectively.

### Effect of norbixin in the preventive supplementation study

**Figure 4:**
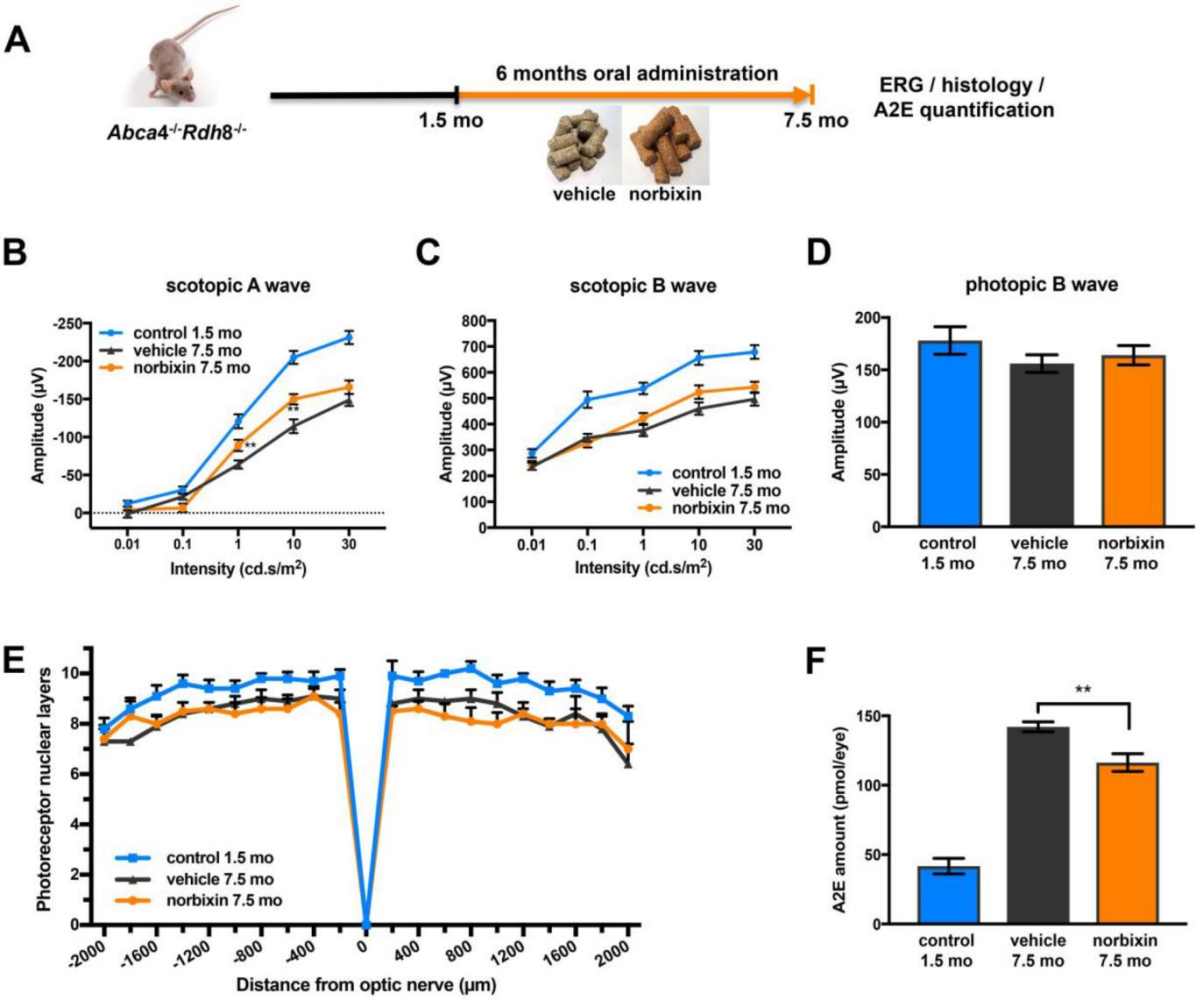
**Effect of norbixin preventive supplementation from 1.5 to 7.5 months in *Abca4^-/-^ Rdh8^-/-^* mice.** (**A**) schematic representation of the 6-month preventive supplementation protocol design. (**B**) Scotopic A wave, (**C**) Scotopic B wave, (**D**) Photopic B wave, recorded after 6 months of oral supplementation with norbixin in *Abca4^-/-^ Rdh8^-/-^* mice compared to mice fed with normal chow (vehicle) and to 1.5-month-old mice. (**E**) Quantification of photoreceptor nuclear layers along the superior and inferior poles of the retina each measured every 200 μm apart from the optic nerve. (**F**) A2E quantification in eyes from 1.5-month-old *Abca4^-/-^ Rdh8^-/-^* mice, 7.5-month-old mice fed with normal chow or with norbixin-containing pellets. Bars represent mean ± s.e.m. with n= 8 mice per group (i.e. n=16 eyes per group for ERG). **p<0.01 compared to vehicle (One-way ANOVA, Dunnett's post-test).

In the preventive supplementation experiment, 1.5-month-old *Abca4^-/-^ Rdh8^-/-^* mice were fed with normal pellets or norbixin-containing pellets for 6 months (Figure 4A). Norbixin concentrations in the eye were below detection limits (data not shown). However, total norbixin concentration including isomers and glucuronide-conjugated forms and norbixin-conjugated glucuronides were detected in the plasma after 6 months of supplementation, confirming the exposure of mice to norbixin administered via food complementation (Table 1). After 3 months of supplementation no difference was measured in scotopic and photopic ERG in mice fed with norbixin-containing pellets or with normal pellets (data not shown). In contrast, after 6 months of supplementation we observed a limited, but significant (*p*<0.01), protection of scotopic A-wave ERG in mice supplemented with norbixin-containing chow compared to mice fed with normal pellets. Indeed, at a 10 cd.s/m^2^ flash intensity (that corresponds to a mixed rod and cone response), in 7.5-month-old mice fed with normal chow, A wave amplitude was reduced by 44.2% compared to the 1.5-month-old mice (Figure 4B) whereas in 7.5-month-old mice fed with norbixin-containing-chow, A wave amplitude was only reduced by 26.8% compared to the 1.5-month-old mice (Figure 4B). Therefore, norbixin pellet administration reduced the loss of scotopic A wave intensity by 60.7% when compared with normal pellets (Figure 4B). However, no significant difference in either scotopic B nor photopic B waves were observed between the two groups of mice (Figure 4C, 4D). Compared to normal chow, norbixin-containing pellet supplementation had no effect on photoreceptor degeneration at 7.5 months (Figure 4E). We observed a dramatic increase in A2E accumulation between 1.5 and 7.5 months in mice fed with normal pellets (Figure 4F). Interestingly, we measured a reduction (18.3 %, *p*<0.01) in A2E accumulation in mice supplemented for 6 months with norbixin (Figure 4F).

**Table 1:** norbixin + isomers + norbixin glucuronide conjugate plasma concentrations.

**Experiment**	**Mean ± SD (nM)**
Preventive	1201 ± 256.5
Early curative	887,4 ± 295.7
Late curative	1782 ± 346.1

### Effect of norbixin in the early curative supplementation study

**Figure 5:**
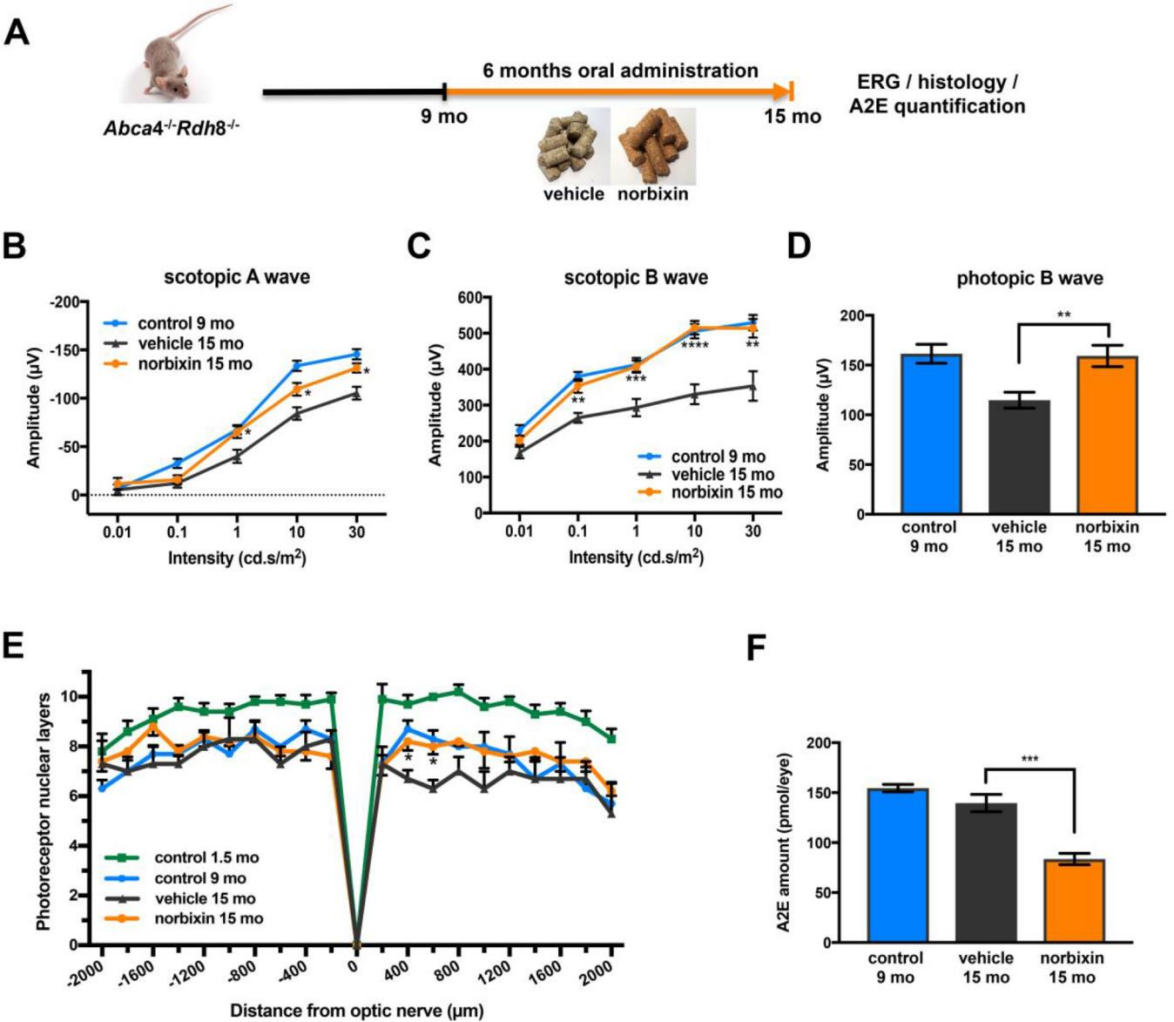
**Effect of norbixin early curative supplementation from 9 to 15 months in *Abca4^-/-^ Rdh8^-/-^* mice.** (**A**) Schematic representation of the 6-month early curative supplementation protocol design. (**B**) Scotopic A wave, (**C**) Scotopic B wave, (**D**) Photopic B wave ERG recorded after 6 months of oral supplementation with norbixin in *Abca4^-/-^Rdh8^-/-^* mice compared to mice fed with normal chow (vehicle) and to 1.5 and 9-month-old mice. (**E**) Quantification of photoreceptor nuclear layers along the superior and inferior poles of the retina each measured every 200 μm apart from the optic nerve. (**F**) A2E quantification in eyes from 9-month-old *Abca4^-/-^ Rdh8^-/-^* mice, 15-month-old mice fed with normal chow or with norbixin-containing pellets. Bars represent mean ± s.e.m. with n = 6 per group (i.e. n=12 eyes for the norbixin treated group and n=11 eyes for the vehicle treated group for ERG). *p<0.05, **p<0.01, ***p<0.001, ****p<0.0001 compared to vehicle (One-way ANOVA, Dunnett's post-test).

In the early curative supplementation experiment, untreated 9-month-old *Abca4^-/-^ Rdh8^-/-^* mice were fed with normal pellets or norbixin-containing pellets for 6 months (Figure 5A). At the beginning of treatment, the retinal function of 9-month-old *Abca4^-/-^Rdh8^-/-^* mice was already decreased by 28.5% and 19.8 % for scotopic A and B waves (flash intensity: 10 cd.s/m^2^), respectively and by 10.3 % for the photopic B wave compared to 2-month-old mice (Figure 2A–2C). At the end of the early curative supplementation experiment (15-month-old mice), norbixin concentration in the eye was again below detection limits (data not shown). Significant amounts of norbixin (including free norbixin and norbixin glucuronide conjugates) were detected in plasma after 6 months of supplementation (Table 1), confirming norbixin exposure. After 3 months of supplementation, we observed no statistical difference between norbixin-treated and control animals in scotopic A and B waves and photopic B wave measured by full-field ERG (data not shown). By contrast, after 6 months of supplementation we observed a strong and significant preservation of scotopic A (Figure 5B) and B waves (Figure 5C), as well as photopic B wave (Figure 5D) in mice supplemented with norbixin compared to mice fed with normal pellets. Most interestingly, the photopic B wave amplitude in 15-month-old mice that were fed with norbixin-containing pellets for 6 months was similar to the photopic B wave amplitude of 9-month-old mice at the beginning of treatment with norbixin (Figure 5D). This result demonstrates that norbixin supplementation fully preserves the function of cone photoreceptors. In 15-month-old mice supplemented during 6 months with norbixin, a statistically significant protection of photoreceptor degeneration was observed in the inferior retina compared to mice fed with normal chow (Figure 5E; *p*<0.05). Consistent with functional protection, A2E accumulation in RPE was strongly reduced (-40 %; *p*<0.001) in mice fed with pellets containing norbixin compared to mice fed with normal pellets (Figure 5F). No difference in lipofuscin granule accumulation was observed following transmission electronic microscopy (TEM) analysis of mice supplemented with norbixin compared to mice fed with normal chow (data not shown). No differences were either noted regarding RPE65 nor cathepsin D expression in the RPE layer of mice treated with norbixin compared to mice fed with normal chow (data not shown). By contrast an increase of Müller cells GFAP staining was observed following supplementation with norbixin (data not shown).

### Effect of norbixin in the late curative supplementation study

**Figure 6:**
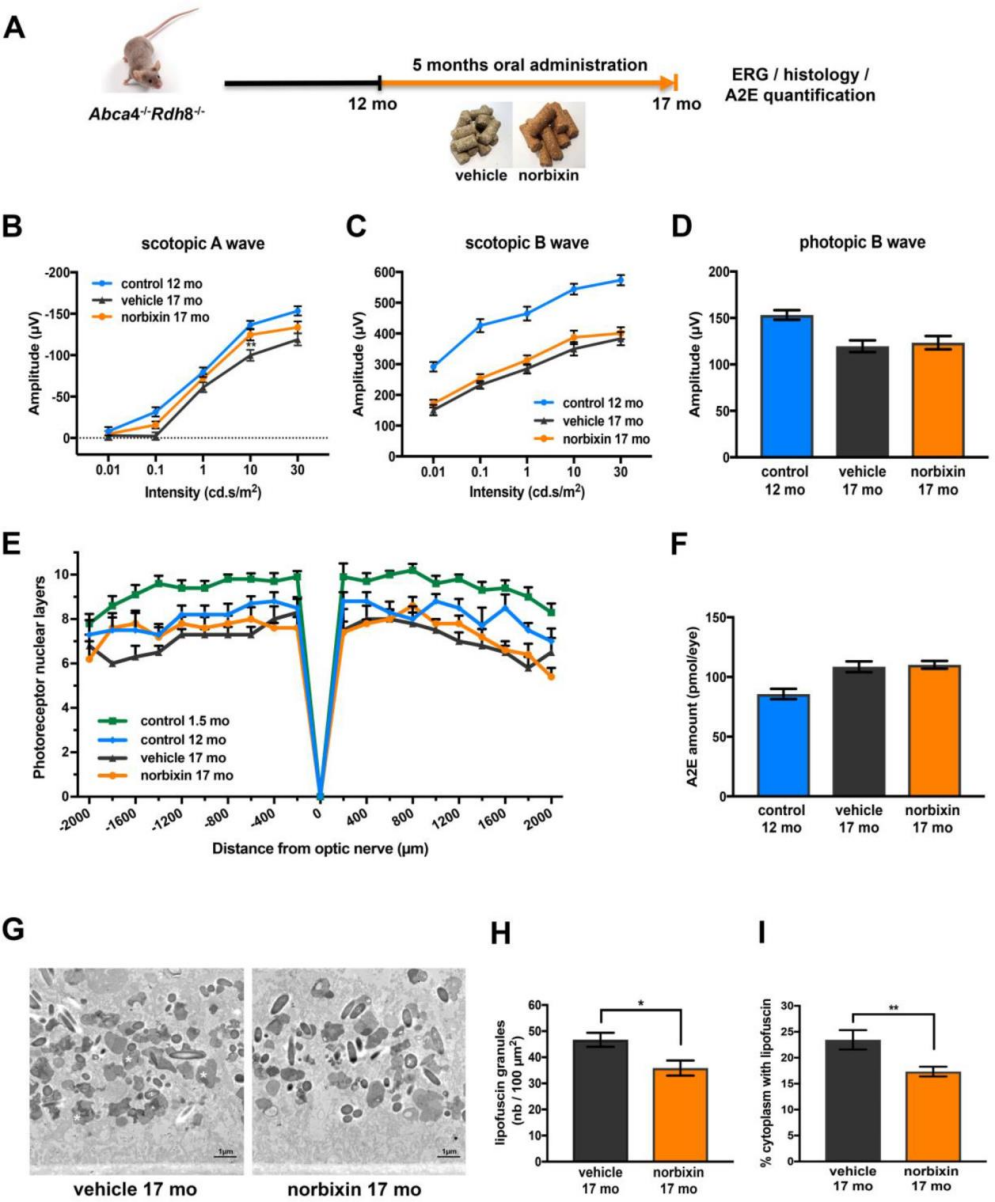
**Effect of norbixin late curative supplementation from 12 to 17 months in *Abca4^-/-^ Rdh8^-/-^* mice.** (**A**) Schematic representation of the 5-month late curative supplementation protocol design. (**B**) Scotopic A wave ERG recorded after 5 months of oral supplementation with norbixin in *Abca4^-/-^ Rdh8^-/-^* mice compared to mice fed with normal chow (vehicle) and to 1.5- and 12-month-old mice. (**C**) Scotopic B wave. (**D**) Photopic B wave. (**E**) Quantification of photoreceptor nuclear layers along the superior and inferior poles of the retina each measured every 200 μm apart from the optic nerve. (**F**) A2E quantification in eyes from 12-month-old *Abca4^-/-^ Rdh8^-/-^* mice, 17-month-old mice fed with normal chow or with norbixin-containing pellets. (**G**) Representative images of lipofuscin content in RPE cells of 17-month-old vehicle and norbixin-treated mice. Large granules of lipofuscin are found in the RPE cytoplasm (white asterisk). (**H**) Histograms showing the quantified lipofuscin granules expressed by area of 100 μm^2^. (**I**) Histograms representing the surface of cytoplasm occupied by lipofuscin and expressed in percentage of total cytoplasm surface. Bars represent mean ± s.e.m. with n = 8 per group (i.e. n=16 eyes per group for ERG). *p<0.05, **p<0.01 compared to vehicle (One-way ANOVA, Dunnett's post-test).

During the late curative supplementation experiment 12-month-old *Abca4^-/-^ Rdh8^-/-^* mice were fed with normal pellets or norbixin-containing pellets for 5 months (Figure 6A). Norbixin was not detected in the eyes of mice fed during 5 months with norbixin-containing pellets (data not shown). Norbixin + norbixin-glucuronide conjugate plasma concentration was approximately 1.5- to 2- times higher than in the preventive and early curative supplementation experiments, respectively (Table 1). Nevertheless, after three months of supplementation, norbixin did not preserve scotopic A and B waves and photopic B wave (data not shown). After 5 months of supplementation with norbixin containing pellets, the loss of scotopic A wave ERG (flash intensity: 10 cd.s/m^2^) was reduced by 67.3 % when compared with ERG of animals that received normal pellets (Figure 6B; *p*<0.01). However, no significant difference in neither scotopic nor photopic B wave ERGs was observed between the two groups of mice (Figure 6C, 6D). No significant difference in photoreceptor degeneration was noted between 17-month-old mice fed with pellets containing norbixin or normal pellets for 5 months (Figure 6E). A2E accumulation in 17-month-old mice did not differ between mice fed during 5 months with normal chow and with norbixin-containing pellets (Figure 6F). Interestingly, the number of lipofuscin granules quantified in RPE cells by TEM was slightly, but significantly, reduced (-23.2 %; *p*<0.05) in 17-month-old mice fed with norbixin-containing pellets compared to same-age mice fed with normal pellets (Figure 6G, 6H). The cytoplasm surface occupied by this material reached 23.4 % in the normal chow-fed mice, whereas it was significantly reduced to 17.3 % (*p*<0.01) in the norbixin-fed mice (Figure 6I).

## DISCUSSION

We have previously shown that 9’-*cis*-norbixin, a 6,6’-di-*apo*-carotenoid extracted from annatto (*Bixa orellana*) seeds, protects primary porcine RPE cells against phototoxicity induced by A2E and blue-light illumination *in vitro* [22]. Norbixin was also shown to reduce the uptake of A2E by primary porcine RPE cells *in vitro* [22]. In addition, 3 months of oral supplementation with norbixin in drinking water reduced the A2E ocular accumulation *in vivo* in *Abca4^-/-^Rdh8^-/-^* mice [22]. In the same mouse model, a single norbixin intraocular injection inhibited retinal degeneration and the loss of full-field ERG, induced following blue-light illumination [22]. In addition, when 50 to 100 mg/mL of norbixin was injected intraperitoneally, almost complete neuroprotection was achieved in albino rats subjected to BLD [22]. In the present study, we tested on one hand, the effect of four intraperitoneal injections of 10 mg/kg of norbixin administered before and during the course of a modified BLD exposure in BALB/c mice, an acute model used to rapidly study the neurodegeneration symptomatic of AMD, and, on the other hand, the effect of norbixin supplementation for 5 to 6 months in *Abca4^-/-^ Rdh8^-/-^* mice of different ages, a chronic model of AMD and STGD.

Here, we demonstrate that norbixin IP injections are partially neuroprotective and protect photoreceptor function following retinal degeneration induced by blue-light exposure. The protective effect of norbixin in the blue-light model is probably associated with its antioxidative properties as previously demonstrated *in vitro* [22, 42] and *in vivo* [43]. Despite the fact that we haven’t measured the specific effect of norbixin on expression of inflammatory markers in retina of Balb/c mice subjected to BLD we quantified sub-retinal infiltration in this model. We observed a small trend toward reduction of macrophages infiltration by norbixin, however this was not statistically significant (data not shown). The absence of a significant effect of norbixin on macrophages infiltration in the BLD model could be explained by the limited dose of norbixin used in this experiment. Indeed, the dose of norbixin only induced a 30 % neuroprotective effect in the retina. It could be hypothesized that a higher dose of norbixin would induce a significant reduction of macrophages infiltration in the sub-retinal space. Further experiments to confirm this hypothesis and to decipher the neuroprotective mechanism of norbixin in this model are therefore still required.

*Abca4^-/-^ Rdh8^-/-^* mice are a model of STGD but also a broader model of retinal degeneration such as AMD [36, 37]. Indeed, both STGD and AMD are characterized by progressive accumulation of A2E and lipofuscin, RPE cell and photoreceptor degeneration eventually leading to the gradual “night” and “day” vision loss that are mediated by rods and cones, respectively [13, 14]. Structural and morphological alterations over time of the retina of *Abca4^-/-^ Rdh8^-/-^* mice has been extensively described [37]. It is noteworthy that despite the fact that the mice we used also carried the *rd8* mutation in the *Crb1* gene, we haven’t observed the formation of photoreceptor “rosettes” in the retina nor focal inferior retinal degeneration characterized by the formation of local retinal folds representative of *rd8^-/-^* mouse retinal phenotype [44] (data not shown). It has been shown that *Crb1^rd8/rd8^* mutation is necessary but not sufficient for the development of these degenerative features and that the influence of small genetic background difference is involved in the resulting phenotype [45]. Therefore, we can expect that our strain does not express gene mutations necessary to induce a degenerative phenotype as it was already the case in our previous publication [22].

In the present study, we describe the kinetics of expression of cathepsin D, RPE65 and GFAP expression and the progressive loss of visual function in relation with photoreceptor degeneration, and of the accumulation of A2E in eyes of *Abca4^-/-^ Rdh8^-/-^* mice as they aged between 2 months and 18 months. Ocular A2E accumulation in the eyes of *Abca4^-/-^ Rdh8^-/-^* mice appeared to be biphasic with a maximum observed at 9 months followed by a plateau afterwards. The rapid increase of ocular A2E concentration has been previously reported in “young” *Abca4^-/-^ Rdh8^-/-^* mice up to the age of 6 months [36]. Interestingly, while retinal degeneration and loss of ERGs appeared constant during aging, we noted that photoreceptor loss and decrease in retinal function was more pronounced between 2 and 6 months and 12 and 18 months than between 9 and 15 months. Evolution of photoreceptor function over time might be related to the kinetics of A2E accumulation during the early phase in young mice and following its stabilization during the late phase in old animals. A2E and other toxic all-*trans*-retinal derivatives accumulate with age in the retina and RPE as by-products of the visual cycle even in non-pathological conditions [46]. In *Abca4^-/-^ Rdh8^-/-^* mice, lacking two proteins essential for avoiding accumulation of all-*trans*-retinal derivatives in photoreceptors and RPE, high amounts of A2E are found in the RPE. Similarly, in the retina of AMD and STGD patients, A2E also accumulates faster and leads to retinal degeneration [46]. A2E promotes the release of Ca^2+^ from the endoplasmic reticulum leading to the production of reactive oxygen species (ROS) by NADPH oxidase [46, 47]. Excessive ROS production and oxidative stress in general contributes to photoreceptor cell death and retinal degeneration [23] and is recognized as a major risk factor for dry AMD development [48–50]. It could be hypothesized that in young *Abca4^-/-^ Rdh8^-/-^* mice, rapid accumulation of toxic A2E is responsible for the early decline in photoreceptor counts and visual function that we observed. Then, approximately around 9 months of age, due to the reduction of the number of photoreceptors and reduction in the visual cycle activation, demonstrated by reduced ERG amplitudes, the production of A2E may be progressively slowed, reaching an equilibrium illustrated by the plateau observed thereafter. During this intermediate period, when A2E is produced at a slower rate, A2E may also be more sensitive to photodegradation. Indeed, due to the reduced number of photoreceptor nuclei, the retina is thinner, allowing increased light intensity to reach A2E internalized in RPE cells [51]. In the meantime, apoptosis of photoreceptors continues at a slower rate, probably secondary to the early phase of photoreceptor degeneration progressively displacing the equilibrium between A2E concentrations and numbers of photoreceptors established at 9 months. It is to note that A2E ocular concentrations measured in our experiments are slightly higher and retinal degeneration slightly milder when compared to results found in the article by Maeda and colleagues [36], but are consistent with reported values by others [52]. Moreover, during the long-term studies presented here we used the same eyes to dose A2E and norbixin. Since norbixin could have been present in ocular fluids and to avoid losing vitreous/aqueous humor, we did not perform dissections of eyes from *Abca4^-/-^ Rdh8^-/-^* mice to determine the exact location of A2E in these mice. However, we assume that A2E is mostly present in the RPE layer. Indeed, in previous dosing experiments performed on mice and dog eyes we noted that A2E is almost exclusively found in the RPE (data not shown). This observation is also consistent with the publication from Lenis et al. [5]. Altogether, the observations that the kinetics of evolution of ERG amplitudes, A2E accumulation and photoreceptor loss were not constant over time, suggest that the efficacy of a systemic treatment might be dependent on pathological course.

In the present study, we attempted to determine the optimal therapeutic window during which oral supplementation with norbixin is the most effective in relation to kinetics of A2E accumulation and of loss of visual function in *Abca4^-/-^ Rdh8^-/-^* mice. Here we report that 6-month oral supplementation of 9-month-old *Abca4^-/-^ Rdh8^-/-^* mice with chow containing norbixin totally preserves cone function and limits loss of function in rods, as well as photoreceptor degeneration and A2E accumulation. By contrast, during norbixin supplementation of younger mice (starting at 1.5 months), we only observed a slight but significant protective effect of norbixin on scotopic A wave ERG and on retinal accumulation of A2E. This might be related to the fact that sharp decrease in scotopic A wave amplitude and an important A2E accumulation occur during the early retinal degeneration phase. The neuroprotective effect of norbixin in *Abca4^-/-^ Rdh8^-/-^* mice during the early curative supplementation confirms our previous observations in acute blue-light induced retinal degeneration model *in vivo* in the same mice [22]. In the three supplementation protocols (preventive, early and late curative), norbixin prevented the loss of scotopic A wave amplitude measured by ERGs. This suggests a protective effect on rod photoreceptors by norbixin. Rods are the first neurons affected during the early stages of AMD and STGD [13, 14]. They are also much more abundant in the mouse retina than cones [13, 14]. In the preventive supplementation the protective effect of norbixin on rod function, was associated with a slight reduction of A2E concentration (Figure 2). This slight reduction however appears insufficient to prevent the loss of cone function as demonstrated by the absence of effect on photopic B wave. The loss of rod’s function is a progressive phenomenon observed even in 15-month-old *Abca4^-/ -^Rdh8^-/-^* mice and was also reduced by norbixin supplementation in the late curative supplementation protocol. It is important to note that the late curative experiment presented here, was performed in female mice rather than male mice in the other protocols. However, we compared ERG results obtained in 17 months male mice treated with norbixin for 5 months (between 12 and 17 months) and ERG amplitudes data obtained in male mice aged 18 months from the experimental group used in Figure 2. We observed a protection of scotopic A wave ERG (but not scotopic B wave) linked with rod function in norbixin complemented male mice (data not shown). Due to the difference of age between the two groups of male mice we cannot conclude that these effects are solely related with norbixin treatment with the exclusion of age, but this observation is consistent with the preferential preservation of rod photoreceptors reported for female mice in the late curative experiment (Figure 5B). Therefore, we are confident that the results reported here on the late curative complementation in female are representative of a phenomenon also occurring in male mice. The protective action of norbixin extended to cone photoreceptor function in mice supplemented between 9 and 15 months of age. Norbixin treatment during the early curative supplementation reduced by 40 % the A2E retinal concentration. This suggests that a sharp reduction of A2E concentration is necessary to protect cone photoreceptor function, whereas a more limited reduction of A2E concentration appears to be sufficient to protect rod photoreceptor function. Other compounds including retinylamine [36, 53], C20-d3-vitamin A, [54], Emixustat [55], primary amines [56], omega-3 fatty acids [57] and the selective oestrogen receptor modulator raloxifene [47], among others, have been shown to reduce A2E accumulation and concomitantly to “rescue” retinal degeneration in *Abca4^-/-^ Rdh8^-/-^* mice or *Abca4^-/-^* *in vivo*. Altogether, this supports the hypothesis that norbixin effectiveness is at least partly linked to the level of reduction of ocular A2E concentration.

The exact mechanism(s) of reduction of A2E concentration *in vitro* and *in vivo* by norbixin remain(s) to be determined. But, based on our ERG results, it seems unlikely that norbixin supplementation limits A2E production *in vivo* by slowing down the visual cycle. We also showed previously *in vitro* that norbixin reduces the amounts of A2E internalized by porcine RPE cells as determined by HPLC-MS/MS [22]. Therefore, norbixin-induced reduction of intraocular A2E concentration in *Abca4^-/-^ Rdh8^-/-^* mice could be due to direct effects on RPE cells *in vivo*. We could also hypothesize that in the BLD experiments reported here, the neuroprotective effect of norbixin observed in *Abca4^-/ -^Rdh8^-/-^* mice *in vivo* may be due to a reduction of oxidative stress and ROS production. Indeed, norbixin has been shown to reduce oxidative stress in rats [42] and humans [43] following a high-fat meal diet. Further experiments to confirm the antioxidant properties of norbixin in the context of retinal degeneration *in vivo* and in RPE cells *in vitro* are still required. In the present article which focuses on the effect of norbixin *in vivo* in mice models of AMD and STGD we haven’t described any mechanistic processes of norbixin. However, experiments *in vitro* on primary porcine RPE cells are actually performed to decipher the potential mechanism of action of norbixin (V. Fontaine et al. in preparation). Indeed, in addition with reduction of A2E accumulation, anti-apoptotic, antioxidant and anti-inflammatory properties of norbixin could play a role in the protection of visual function and in the neuroprotective effect of norbixin that we report in the present study *in vivo*. In order to determine if norbixin displayed an anti-inflammatory effect in the late curative experiment we quantified macrophages infiltration in the subretinal space of *Abca4^-/-^ Rdh8^-/-^* mice (data not shown) *in vivo* but we did not observe any significative differences. It has been proposed that A2E may potentiate subretinal macrophage accumulation [58]. Therefore, the slight reduction of A2E accumulation obtained in our model by norbixin may not be sufficient to significantly reduce macrophage recruitment. However, it could be hypothesized that norbixin may alter macrophage activation or differentiation *in vivo*. This will be the aim of future experiments. In order to better understand the effects of norbixin in the retina *in vivo* and to determine whether norbixin effects target specifically the retina or RPE or both, we performed GFAP, RPE65 and cathepsin D stainings on retinal sections of eyes obtained from *Abca4^-/-^ Rdh8^-/-^* mice treated with norbixin versus untreated in the early curative supplementation experiment. However, we did not observe any differences if RPE65 nor cathepsin D expression in both groups. Surprisingly, we observed enhanced GFAP staining of Müller cells in the retina of norbixin supplemented mice. Further experiments are required to decipher the mechanisms behind the protective effects of norbixin.

To our knowledge, this is the first report demonstrating the efficiency of treatment given orally through food complementation on the photoreceptor function in the aging *Abca4^-/-^Rdh8^-/-^* mouse model. Most of the previous studies performed to measure the efficacy of molecules on the retinal function, using this *Abca4^-/-^ Rdh8^-/-^* mouse model, were done by intraperitoneal or intravitreal injections and after light damage in young mice [36, 47, 53, 56, 59–61]. Maeda and colleagues also showed the beneficial effect of 9*-cis*-retinal administrated by monthly oral gavage over 6 or 10 months in 4-month-old C57BL/6 mice on scotopic A and B waves, as well as flicker ERG [62]. Similar observations were reported following weekly gavage for one month in 12-month-old *Rdh5^-/-^Rdh11^-/-^* mice [63]. In another study, the same group showed that 3 months treatment with primary amines in 1-month-old *Abca4^-/-^ Rdh8^-/-^* mice force-fed daily reduced A2E accumulation, photoreceptor degeneration, and retinal functions [56].

Based on our previous results showing that norbixin increases RPE survival and reduces A2E internalization *in vitro* [22], we assume that norbixin’s neuroprotective effect is local. However, free norbixin concentrations in the eyes were below detection limits in all supplementation experiments. It could be hypothesized that limited amounts of free norbixin present in the plasma access the eye and are consumed in a continuous fashion. Oral supplementation exposure of norbixin in mice depends on the quantity of food absorbed by each mouse and also depends on the time of day of the analysis of plasma concentrations relative to food consumption which occurs mainly at night time. Therefore, we cannot assume that all mice received the same amount of norbixin during supplementations. Nevertheless, at 3 and 6 months, norbixin was detected in the plasma of all supplemented mice across all experimental designs, confirming the exposure of mice to norbixin administered via food as early as 3 months. Interestingly, in the early curative supplementation experiment, plasma norbixin and norbixin glucuronide conjugate concentration was the lowest. Nevertheless, the observed partial neuroprotection, and full preservation of scotopic and photopic ERG amplitudes, suggest that this level of plasma exposure is sufficient to support norbixin biological effects. As a mean, we calculated that norbixin consumption represents approximately a daily intake of 47.5 mg +/- 5 mg per kg/mouse. This value is in the same range of administration in the BLD study, where mice received four intraperitoneal injections of 10 mg/kg each. Nevertheless, we have reported previously in acute BLD model in albino rats a more profound effect of our tested compound. The partial efficacy reported here in acute and long-term models of retinal degeneration may be due to the small doses used and might be improved by increasing the doses of norbixin administered. Increasing bio-availability of norbixin into the eye following systemic administration may also improve efficacy and we are actually working on these two options.

In conclusion, our present study demonstrates that systemic administration of norbixin in the acute BLD model of dry AMD is neuroprotective and partially preserves photoreceptor function. In addition, 6 months of oral supplementation with norbixin is effective in *Abca4^-/-^Rdh8^-/-^* mice. We show that chronic norbixin supplementation reduces the concentration of A2E in the eye, that norbixin is neuroprotective, and preserves visual function of *Abca4^-/-^Rdh8^-/-^* mice, modelling retinal degenerative conditions such as STGD and dry AMD. We believe that treatment using norbixin could potentially preserve “night” and “day” visual acuity in humans affected by dry AMD and STGD. It is essential for patient care to develop drugs that are effective on visual function following oral administration rather than by repeated local intraocular injections. These results demonstrated the effectiveness of the norbixin in a chronic and acute model of retinal degeneration and could offer a new therapeutic strategy, alone or in combination with gene therapies, for AMD and/or STGD patients. Thus, norbixin is a good drug candidate to treat patients and may provide a cure for these very debilitating diseases.

## MATERIALS AND METHODS

### Ethics statement

All procedures were carried out according to the guidelines on the ethical use of animals from the European Community Council Directive (86/609/EEC) and were approved by the French Ministry of Agriculture (OGM agreement 6193) and by the Committee on the Ethics of Animal Experiments of the French Ministry of Research. All efforts were made to minimize suffering.

### Animals

BALB/c mice were provided by Envigo (Gannat, France). Pigmented *Abca4^-/-^ Rdh8^-/-^* mice carrying the Rpe65-Leu450 mutation and the *rd8* mutation in the *Crb1* gene were obtained from Case Western Reserve University [36]. All animals were housed under 12-hour on/off cyclic normal lighting.

### Reagents/chemicals

All general chemicals were from Sigma (St. Louis, MO, USA). Reagents for cell culture and Alexa Fluor® 488 **-** conjugated secondary antibodies were from Thermo Fisher Scientific (Waltham, MA, USA). TrueBlack® was from Biotium (Fremont, CA, USA). Goat anti-human Cathepsin D antibody was from Santa Cruz Biotechnology (Dallas, TX, USA), mouse anti-bovine RPE65 antibody and rabbit anti-human GFAP were from Abcam (Cambridge, UK). Ketamine, xylazine, tropicamide and oxybuprocaine chlorhydrate were from Centravet (Maison-Alfort, France). Lubrithal eye gel was from Dechra Pharmaceuticals (Northwich, UK). Optimal cutting temperature compound and other reagents for histology were from Roth Sochiel (Lauterbourg, France). Agar 100 resin kit was from Agar Scientific (Stansted, UK). 9'-*cis*-Norbixin was prepared from 9'-*cis*-bixin (AICABIX P, purity 92 %) purchased from Aica-Color (Cusco, Peru) upon alkaline hydrolysis as previously described [22] and according to Santos et al. [64]. The obtained product (the 9'-*cis* isomer) showed an HPLC purity of 97 % as confirmed by ^1^H-nuclear magnetic resonance (using malonic acid as internal standard). Fresh solutions of 9'-*cis*-norbixin, stored as powder at -80°C, were prepared in DMSO.

### Immunohistochemistry

Retinal cryosections of *Abca4^-/-^ Rdh8^-/-^* mice aged 2 and 18 months were permeabilized with Triton X100 (0.05% in PBS; 5 min at RT) and saturated with NGS (10% in PBS) or BSA (3% in PBS) during 1 h at RT. For cathepsin D immunostaining sections were depigmented in H_2_O_2_ (3% in PBS) during 24 h before staining. Primary antibodies against GFAP, RPE65 and cathepsin D were diluted in 2% NGS or 1% BSA and incubated over night at 4°C and followed by Alexa Fluor® 488 **-** conjugated secondary antibodies during 1 h at RT. In order to quench lipofuscin auto-fluorescence in RPE a final incubation with TrueBlack® was performed. Sections were stained with Hoechst 33342 to label nuclei and representative pictures were taken using a fluorescence microscope (Nikon TiE) equipped with a CoolSNAP HQ2 camera. For each age retinal cryosections from 3 different mice were used.

### Synthesis of A2E and A2E-Propylamine

A2E (*N*-retinylidene-*N*-retinylethanolamine) was synthesized by Orga-link (Magny-Les- Hameaux, France) as described before [65]. Briefly, all-*trans*-retinal, ethanolamine and acetic acid were mixed in absolute ethanol in darkness at room temperature over 7 days. The crude product was purified by preparative HPLC in the dark to isolate A2E with a purity of 98 % as determined by HPLC. A2E (20 mM in DMSO under argon) was stored at -20°C. A2E-propylamine (an analogue of A2E) was synthesized as previously described [22] using propylamine instead of ethanolamine.

### Intraperitoneal treatment and blue-light damage (BLD)

Four groups of 8 BALB/c mice were used for this study. Mice were injected intraperitoneally with either norbixin (10 mg/kg in 5 % Tween 80 in PBS), or an equivalent volume of vehicle (5 % Tween 80 in PBS) 30 min prior to light damage and 1, 2.5 and 4 hours after the beginning of the exposure. A custom-made light-damage device equipped with fluorescent lamps (Phillips TL-D 36W/18) with UV filter was used to induce BLD in mice (Durand, St-Clair de la Tour, France). All manipulations with the animals were performed in dim red light. Pupils were dilated with 1 % atropine eye solution before illumination. Mice, previously maintained in a 12-hour light (≈ 10 lux)/ 12-hour dark cycle environment for two weeks, were dark-adapted for 24 hours and light damage was induced at 4000 lux for 4 hours. Following exposure to light damage, animals were placed in the dark for 24 hours and then returned to the dim cyclic light environment for 7 days. Two control groups were used: i) non-injected and illuminated mice and ii) non-injected and non-illuminated mice.

### Kinetics of ERGs, photoreceptor loss and A2E accumulation in *Abca4^-/-^ Rdh8^-/-^* mice

A total of 300 *Abca4^-/-^ Rdh8^-/-^* male and female mice of different ages (2, 4, 6, 9, 12, 15 and 18 months) were used in order to perform a kinetic analysis of ERGs, photoreceptor loss and A2E accumulation.

### Norbixin-containing pellet preparation preservation and consumption

Custom rodent diet was formulated and irradiated (25 kGy) by Special Diet Services (Witham, UK). Norbixin (600 μg/g) was incorporated in 10 mm RM1 compression pellets. The pellets were stored at -20°C until use and were administered as standard diet (*ad libitum*). The concentration of norbixin in the pellets at the end of each batch was determined by HPLC MS/MS. The mean concentration was 377.9 μg/g +/- 40.93 μg/g. Based on the norbixin pellet concentration, we calculated that male mice weighing 42.1 g after 3 months supplementation consumed 5.3 g of pellets every day, which correspond to a daily dose of 47.5 mg +/- 5 mg per kg.

### *In vivo* norbixin supplementation

In order to test the preventive/curative actions of oral norbixin against retinal neurodegeneration a total of 54 *Abca4^-/-^ Rdh8^-/-^* mice of different ages were used. In the first “preventive study”, two groups of 8 males aged 1.5 months received control chow or chow containing norbixin orally for 6 months. In a second experiment “early curative study” two groups of 6 males aged 9 months received norbixin mixed with chow or control chow orally for 6 months. In a third experiment “late curative study”, two groups of 8 females aged 12 months received norbixin mixed with chow or control chow orally for 5 months. In each experiment full-field ERG was performed after 3 months of supplementation. After 5 or 6 months of supplementation, ERG was measured in both eyes (n=16 per group in the preventive and late curative experiments and n=12 for norbixin treated group and n=11 in the vehicle group of the early curative study). Blood was collected by cardiac puncture in all mice before being euthanized. In each group half of the eyes were removed for A2E and norbixin measurements and half of the eyes were used for histological analyses.

### Full-field electroretinogram

ERG recordings were performed with the Espion visual electrophysiology system (Diagnosys LLC, Lowell, MA, USA) that includes a ColorDome Ganzfeld. ERG was performed one week after BLD. After overnight dark adaptation, mice were anesthetized with ketamine (100 mg/kg) and xylazine (10 mg/kg). Eye drops were used to dilate the pupils (0.5 % tropicamide+ 5% phenylephrine hydrochloride) and anesthetize the cornea (0.4 % oxybuprocaine chlorhydrate). Body temperature was maintained at 37°C using a circulating hot-water heating pad. Corneal electrodes (Ocuscience, a subsidiary of Xenolec Inc., USA) were placed on the corneal surface of each eye. Lubrithal eye gel was used to maintain good contact and corneal moisture. Needle electrodes placed subcutaneously in cheeks served as reference and a needle electrode placed in the back served as earth. The ERG was recorded from both eyes simultaneously after placing the animal into the Ganzfeld bowl. Five responses to light stimulus at increasing intensities (0.01, 0.1, 1, 10 and 30 cd.s.m^-2^) were averaged for scotopic response. After 5 min of light adaptation, the photopic response was recorded at the highest stimulus (average of 5 measurements at 30 cd.s.m^-2^).

### Histology and photoreceptor counting

Following ERG measures, mice were euthanized and eyes were enucleated and dissected to remove the cornea and lens. For cryosection, eyes were fixed in 4 % paraformaldehyde/ 5 % sucrose (in PBS) for one hour at 4°C. The eye cups were then cryoprotected by successive bathing in 5 % sucrose (1h), 10 % sucrose (1h) and 20 % sucrose (overnight), embedded in optimal cutting temperature compound, and cryosections (10 μm) were prepared using Superfrost® Plus slides and stored at -20°C until analysis. Sections were stained with Hoechst 33342 to label nuclei and were scanned using a nanozoomer (NDP.scan v2.5.86, Hamamatsu, Japan) with fluorescence imaging modules. Photoreceptor nuclei were quantified at 200 μm intervals superior and inferior to the edge of the optic nerve head along the vertical meridian using the NDP.view software.

### Electronic microscopy analysis

After enucleation and anterior segment removal, eye cups were fixed in 1.5 % glutaraldehyde and 1 % paraformaldehyde diluted in 0.1 M sodium cacodylate. One mm^2^ sections of eye cups were cut and incubated for 1 h in 1 % osmium tetroxide. Cells were dehydrated through graded concentrations of ethanol (50-70-96-100 %) and infiltrated in epoxy resin (Agar 100 resin kit) at room temperature according to the manufacturer’s instructions and polymerized for 48 h at 60 °C. Ultrathin sections (70 nm) were cut with an ultramicrotome (Ultracut, Leica Microsystems) and collected on 200 Mesh copper grids (EMS). Observations were made with a Field Emission Scanning Electron Microscope (Gemini 500, Zeiss). Lipofuscin quantification was carried out with Fiji software using the «Cell Counter» plugin. Three different images of RPE cells were counted for each eye in each group. The total area of RPE cytoplasm was systematically quantified. Nuclei areas were excluded from the measure. Results are expressed as number of lipofuscin granules / 100 μm^2^ of cytoplasm and as the cytoplasmic volume occupied by lipofuscin.

### A2E measurement by HPLC-MS/MS

HPLC-MS/MS analysis was performed on an Agilent 1100 in-line triple quadrupole mass spectrometer (API365 or API3200, Applied Biosystems, Les Ulis, France) operated in MRM positive-ion mode. A2E was eluted on a reverse-phase C18 column (2.1x50 mm; 3.5 μm particle size; Symmetry, Waters, Guyancourt, France) with the following gradient of acetonitrile in water (containing 0.1 % formic acid): 65 to 100 % (4 min), 100 % (5 min), (flow-rate: 0.3 mL/min). A2E-propylamine (25 ng) was used as internal standard. The AUC of A2E and A2E-propylamine were determined in MRM mode with precursor ion/product ion settings, A2E (m/z 592.5/105.1) and A2E-propylamine (m/z 590.6/186.2). For A2E quantification, a calibration curve was performed using various concentrations of A2E (5 to 10000 nM). A2E-propylamine was used as internal standard for A2E quantification by HPLC coupled with tandem mass spectrometry (HPLC-MS/MS).

### A2E measurement in mice eyes

A2E present in eyes was determined with the HPLC-MS/MS method described above. Each eye was homogenized in CHCl_3_/MeOH (1:1, v/v) (0.5 mL) with homogenizer (Precellys-24) during 2 cycles (30 s) at 6500 rpm. The internal standard (A2E-propylamine) was added and the organic layer was extracted. The homogenate was then extracted two times with CHCl_3_/ CH_2_Cl_2_ (0.5 mL). The combined organic extracts were dried *in vacuo* without heating (EZ2, Genevac Ltd Ipswich, U.K.). Then they were dissolved in 100 μL DMSO/MeOH (1:1, v/v) and transferred to microtitre plates. The calibration curve of A2E was prepared in CHCl_3_/MeOH (1:1, v/v) and dried *in vacuo* without heating (EZ2, Genevac), then dissolved in 100 μL DMSO/ MeOH (1:1, v/v). Under these conditions, with an injection volume of 10 μL, the limit of quantification (LOQ) was 10 nM.

### Norbixin concentration determination in pellets, mice plasma and eye samples

HPLC analysis was performed on an Agilent 1200 with DAD. Norbixin was eluted from a reverse-phase C18 column (2.1x50 mm; 5 μm particles; Purospher Star, Merck, Molsheim, France) with the following gradient of acetonitrile in water (containing 0.1 % formic acid): 0 to 90 % (1.5 min), 90 % (1 min), (flow-rate: 0.5 mL/min) and they were monitored at 460 nm. For quantification of norbixin, a calibration curve was performed under the same conditions as the sample matrix, with various amounts of norbixin (10 to 50000 ng/mL). Plasma samples (30 μL) from different animals, and methanol (100 μL) were distributed in a 96-well microtitre plate, mixed for 10 min and precipitated. The microtiter plate was frozen at -20°C for 30 min, thawed and then centrifuged. The hydro-alcoholic phase was removed from each well and transferred into another microtitre plate for LC-MS/MS analysis. Under these conditions, with 20 μL injections, the limit of quantification (LOQ) was 50 ng/mL (= *ca*. 2.5 pmol). Eye samples were treated with the same protocol as for A2E measurements (see above). Norbixin isomers were analyzed by LC-MS/MS on an Agilent 1200 with DAD and in-line triple quadrupole mass spectrometer (6420, Agilent, Les Ulis, France) operated in MRM positive-ion mode. HPLC used a reverse-phase C18-column (2.1x50 mm; Fortis-18) eluted with the following gradient of acetonitrile in water (containing 0.1 % formic acid): 60 to 95 % (2.5 min), 95 % (2 min), (flow-rate: 0.3 mL/min). Norbixin and its isomers or conjugates (= glucuronides) were monitored at 460 nm and MRM mode with precursor ion/product ion ratio (m/z 381.1/144.9).

### Statistical analyses

For statistical analyses, one-way ANOVA followed by Dunnett's tests were performed using Prism 7 (GraphPad Software, La Jolla, CA, USA) depending of the sample size.
